# The influence of ionic strength on carbonate-based spectroscopic barometry for aqueous fluids: an *in-situ* Raman study on Na_2_CO_3_-NaCl solutions

**DOI:** 10.1038/srep39088

**Published:** 2016-12-16

**Authors:** Jia Wu, Shixia Wang, Haifei Zheng

**Affiliations:** 1 State Key Laboratory of Petroleum Resources and Prospecting − College of Geoscience, China University of Petroleum, Beijing, 102249, PR China; 2 College of Science, University of Shanghai for Science and Technology, Shanghai, 200093, PR China; 3 Key Laboratory of Orogenic Belts and Crustal Evolution, Department of Geology, Peking University, Beijing, 100871, PR China

## Abstract

The Raman wavenumber of the symmetric stretching vibration of carbonate ion (*ν*_1_*-*CO_3_^2−^) was measured in three aqueous solutions containing 2.0 mol·L^−1^ Na_2_CO_3_ and 0.20, 0.42, or 0.92 mol·L^−1^ NaCl, respectively, from 122 to 1538 MPa at 22 °C using a moissanite anvil cell. The *ν*_1_ Raman signal linearly shifted to higher wavenumbers with increasing pressure. Most importantly, the slope of *ν*_1_*-*CO_3_^2−^ Raman frequency shift (∂*ν*_1_/∂*P*)_*I*_ was independent of NaCl concentration. Moreover, elevated ionic strength was found to shift the apparent outline of the carbonate peak toward low wavenumbers, possibly by increasing the proportion of the contact ion pair NaCO_3_^−^. Further investigations revealed no cross-interaction between the pressure effect and the ionic strength effect on the Raman spectra, possibly because the distribution of different ion-pair species in the carbonate equilibrium was largely pressure-independent. These results suggested that the ionic strength should be incorporated as an additional constraint for measuring the internal pressure of various solution-based systems. Combining the *ν*_1_*-*CO_3_^2−^ Raman frequency slope with the pressure herein with the values for the temperature or the ionic strength dependencies determined from previous studies, we developed an empirical equation that can be used to estimate the pressure of carbonate-bearing aqueous solutions.

Raman spectroscopy is widely employed for pressure determination in various natural^1^ or artificial environments^2^,^3^ and is often coupled with hydrothermal diamond anvil cell (HDAC) in studies on high *P*−*T* geological systems. In HDAC experiments, the pressure inside the sample chamber is usually determined by measuring the wavenumber shift of an internal ruby-4 or quartz-based^5^ calibrant. However, pressure over the critical point or the presence of water could trigger phase transition or dissolution of the crystal pressure gauge, making mineral spectroscopy infeasible^6^. To address these problems, the use of a solute-based barometer has been proposed. Similar to those of solid minerals, Raman vibrational modes of anions in aqueous solutions are also affected by changes in temperature and/or pressure. The Raman wavenumber of pure liquid water at room temperature has been reported to decline with elevated pressure at a slope (∂*ν*_3244_/∂*P*)_*T*_ of −30.4 cm^−1^ ∙ GPa^−1^ 7. The Raman shift of the symmetric stretching of SO_4_^2−^ in 1 mol·L^−1^ (abbreviated as M, hereinafter) Na_2_SO_4_ solution has been shown to be pressure- and temperature-dependent, which can be described by the *P*−*T* equation of *P* = 190.44 ∙ (∆*ν*_981_)_*P*_ + 0.0027 ∙ *T*^2^ + 2.9019 ∙ *T*−111.68^8^. Schmidt^9^ reported similar behaviors of sulfate ion under high *P-T* conditions in HDAC. The same sulfate-based barometer was used to estimate the internal pressure of a pure sulfate solution in a HDAC experiment^10^. The findings of these studies collectively indicated that pressure measurement based on the Raman shifts of solutes could yield very similar results as conventional methods that rely on spectroscopic characterization of crystal pressure gauges.

Carbonate-bearing hydrothermal fluids are widely present in subduction zones or sedimentary basins^11^,^12^. Because of this, carbonate-based solutions are frequently used as a close approximant of H_2_O and CO_2_ mixed fluids in Raman spectroscopyic studies on geological phenomena under high *P-T* conditions. The shift of the *ν*_1_-CO_3_^2−^ Raman line in aqueous solution toward higher wavenumbers with increasing pressure or decreasing temperature has been well documented by several research groups, including Wu and Zheng^13^ and Schmidt^14^. Frantz^15^ demonstrated that the speciations of both K_2_CO_3_ and KHCO_3_ solutions varied with temperature by analyzing the characteristic Raman peaks of aqueous CO_3_^2−^ and HCO_3_^−^ up to 200 MPa and 550 °C. Although these results hinted at the potential value of using the characteristic Raman peak of CO_3_^2−^ as an alternative barometer for determining the pressure of a hydrostatic system, it should be pointed out that carbonate species, in their native geological environments, almost always coexist with a high level of Cl^−^ and various other electrolytes. Their interactions could influence the Raman lines of other Raman-active species^16^. Based on this principle, Sun, *et al*.^17^ developed a calibration equation to estimate salt concentrations in aqueous solutions. A noticeable disadvantage of this approach is that solutes such as CO_3_^2−^ or SO_4_^2−^ can also modify the OH stretching bands of H_2_O, resulting in an overestimation of Cl^−^18. Moreover, Raman spectra of Na_2_CO_3_ in aqueous solutions could be modified by the concentration of different electrolytes^19^,^20^. Taken together, these results suggested that further studies on the relationship between Raman shift and solution composition would be necessary to enable Raman shifts of solutes as a reliable barometer.

Although there is mounting evidence indicating that the Raman shifts of CO_3_^2−^ are affected by a combination of environmental factors such as pressure, temperature, and salt concentration, no systematic studies have been conducted. Herein we report the *in-situ* Raman spectroscopic measurement of several Na_2_CO_3_-NaCl solution systems at high pressure levels (up to 1538 MPa) and ambient temperature (22 °C). The aim of this study is to examine the dependence of the Raman shift of CO_3_^2−^ on pressure in the presence of different concentrations of Cl^−^ and to develop a pressure equation.

## Results

**Figure 1:**
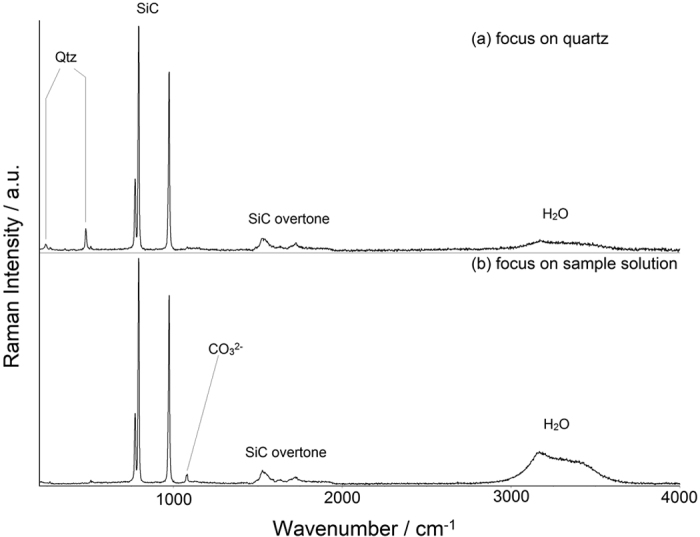
Representative Raman spectra of the pressure gauge (quartz) and the solution samples in the moissanite anvil cell. The scanned region spans the wavenumber range of 50–4000 cm^−1^. (**a**) Laser beam is focused on the quartz. Raman signals at 206 and 464 cm^−1^ are attributed to quartz (Qtz); (**b**) Laser beam is focused on the Na_2_CO_3_-NaCl-H_2_O solution. Raman signals assigned to the sample and moissanite anvil are respectively annotated. The Raman wavenumber of *v*_1_-CO_3_^2−^ in the solution is around 1066 cm^−1^. The Raman band of water covers the region from 3000 to 3700 cm^−1^. The moissanite signals include the shifts at 768 cm^−1^, 789 cm^−1^, and 967 cm^−1^, as well as their overtone bands from 1400 to 1900 cm^−1^.

Because Raman scattering intensity of dispersed solute molecules is inherently weaker than that of crystals composed of the same compound, a high concentration of Na_2_CO_3_ was necessary to generate sufficiently strong Raman signals. We therefore set the concentration of Na_2_CO_3_ at 2 M, which was recommended by earlier studies^21^. Figure 1 shows the combined Raman spectra of the quartz pressure sensor and the sample solution in the wavenumber range of 50 to 4000 cm^−1^. The moissanite anvil was responsible for the strongest peaks located at 768, 789, and 967 cm^−1^ as well as the overtone bands from 1400 to 1900 cm^−1^ 22, whereas quartz showed characteristic Raman lines at 206 and 464 cm^−1^, with the latter being more intense. The single Raman peak at 1066 cm^−1^ was assigned to the symmetric stretching mode of carbonate ions^19^, and the broad band from 3000 to 3700 cm^−1^ was attributable to the H–O vibration of water molecules^23^,^24^.

**Figure 2:**
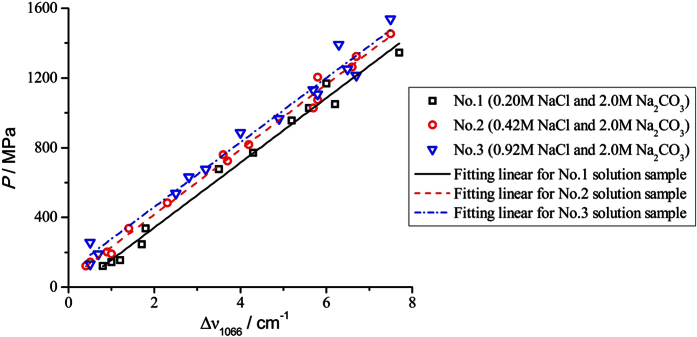
Pressures are plotted against the corresponding *v*_1_-CO_3_^2−^ Raman shifts determined in this study. Three Na_2_CO_3_-NaCl solution samples are annotated as follows: No. 1: 0.20 M NaCl and 2.0 M Na_2_CO_3_, black squares, solid line; No. 2: 0.42 M NaCl and 2.0 M Na_2_CO_3_, red circles, dashed line; No. 3: 0.92 M NaCl and 2.0 M Na_2_CO_3_, blue triangles, dot-dashed line. All three samples are shown to have nearly identical pressure slopes for the *ν*_1_*-*CO_3_^2−^ Raman frequency. In addition, the Raman line shifts to lower frequencies as the concentration of NaCl increases.

For each solution, the internal pressure of the sample chamber was calculated from the Raman frequency shift of the 464-cm^−1^ line of quartz (∆*ν*_464_) using Eq. (3) (Table 1). The pressure was then plotted against the *ν*_1_*-*CO_3_^2−^ Raman shift (∆*ν*_1066_) (Fig. 2). Regression analysis revealed a statistically significant linear relationship between pressure and ∆*ν*_1066_ for each Na_2_CO_3_-NaCl solution (Table 2). The confidence interval was greater than 95% for all the analyses.

## Discussion

The results demonstrated a positive correlation between the Raman wavenumber of CO_3_^2−^ and pressure at ambient temperature for the Na_2_CO_3_-NaCl-H_2_O system (Fig. 2), which was consistent with previous findings obtained from similar high-pressure studies on solutions^14^,^21^, liquid organic matters^25^,^26^, and crystals^27^,^28^. The increase of the Raman frequencies at high pressure is primarily due to the lowered distance between molecules/ions and augmented force constants of various chemical bonds. Both this study and our previous investigation of the binary Na_2_CO_3_-H_2_O system unveiled a positive correlation between pressure and the frequency of the *ν*_1_-CO_3_^2−^ Raman line with a slope (∂*ν*_1_/∂*P*)_*T*_ of around 5.4 cm^−1^ ∙ GPa^−1^ at ambient temperature. It should be noted that several earlier studies on the correlations between pressure and the wavenumber of the *ν*_1_ Raman line reported slightly different (∂*ν*_1_/∂*P*)_*T*_values (unit: cm^−1^ ∙ GPa^−1^; 4.64^21^, 5.06^15^, 5.74^13^). Similar variation was also found for the pressure dependence of the *v*_1_-CH_4_ Raman shift, which was summarized in details by Lu, *et al*.^29^. The discrepancy could be attributed to several possible factors, including pressure determination, Raman spectrograph calibration, and Raman shift of CH_4_ under ambient *P-T* condition. On the other hand, the (∂*ν*_1_/∂*P*)_*T*_value of the *ν*_1_-CO_3_^2−^ Raman line is not only similar to that of the symmetric stretching vibration of sulfate in Na_2_SO_4_ solutions (1.54 M)^9^, but also roughly on the same level as the pressure-dependent Raman frequency shift of commonly used crystal-based sensors such as ^13^C diamond (2.8 cm^−1^ ∙ GPa^−1^) and quartz (9 cm^−1^ ∙ GPa^−1^). Based on Mantegazzi *et al*.’s^10^ previous success in employing sulfate ion as a spectroscopic pressure sensor, we reasoned that aqueous carbonate ion could also serve as a barometer in similar systems.

Several groups have measured the wavenumber of the *ν*_1_-CO_3_^2−^ Raman line at varying concentrations. In order to simplify the expression for different cation species-bearing systems, we used ionic strength to describe the influence of fluid composition on the *ν*_1_-CO_3_^2−^ Raman line. According to the Debye-Hückel theory of electrolytes, the total ionic strength (*I*) of a given aqueous solution can be calculated by the following equation:


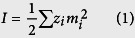


where z_*i*_ and m_*i*_ represent the concentration and charge of ion *I*, respectively. We opted for Eq. (1) as an approximate method for estimating the ionic strength of the Na_2_CO_3_-NaCl solutions in our study.

In Oliver and Davis^19^’s study of K_2_CO_3_ solutions from 1 to 8 M, the frequency was found to decline at a slope (∂*ν*_1_/∂*I*)_*P,T*_ of −0.24 cm^−1^ ∙ M^−1^ (R^2^ = 0.987) with regard to ionic strength. Consistent with these findings, Sun and Qin^30^ reported that the (∂*ν*_1_/∂*I*)_*P,T*_ slope for Na_2_CO_3_ solutions up to 15 wt% (1.556 M, *I* = 4.668 M) and K_2_CO_3_ solutions up to 50 wt% (5.125 M, *I* = 15.374 M) were −0.062 cm^−1^ ∙ M^−1^ (R^2^ = 0.993) and −0.181 cm^−1^ ∙ M^−1^ (R^2^ = 0.998), respectively (Table 3). On the other hand, the influence of ionic strength on Raman frequency might explain the slight variation that we previously observed among the three *P*-∆*ν*_1066_ equations derived from different Na_2_CO_3_ solutions^13^. There is substantial evidence to suggest that the fitting method we implemented should carry a theoretical uncertainty no greater than 0.15 cm^−1^, which is considerably smaller than the actual difference among the three fitting equations^31^,^32^.

There have also been efforts to determine the impact of coexisting ions, such as OH^−^ and Cl^−^, on the *ν*_1_-CO_3_^2−^ Raman spectrum^20^. These results were in satisfactory agreement with our experimental data. The *ν*_1_-CO_3_^2−^ Raman line at 0.100 M was indicated to shift to low frequency in the presence of NaOH, following a quadratic equation of *ν*_1_ = −0.022*I*^2^–0.058*I* + 1065.5 (R^2^ = 0.996). A similar trend was confirmed for 1.0 M Na_2_CO_3_ solutions when NaCl was added to a final concentration of 0.5 − 2.0 M; however, the decline of Raman wavenumber in this case exhibited a linear correlation with the Cl^−^ concentration at a slope (∂*ν*_1_/∂*I*)_*P,T*_ of −0.113 cm^−1^ ∙ M^−1^ 20, or −0.167 cm^−1^ ∙ M^−1^ 30.

At low ionic strength, the interaction network of CO_3_^2−^ in aqueous solution is dominated by its hydrogen bonding with solvent waters. Since the strength of the hydrogen bond only depends on the chemical nature of the bonding molecules, the Raman shift of CO_3_^2−^ would remain relatively constant at low concentrations (e.g., 0.01 M and 0.1 M)^19^. As the concentration of CO_3_^2−^ rises, however, electrostatic interactions between CO_3_^2−^ and the surrounding cations would become more significant. The increase in the density of cations would encourage the formation of contact ion pairs (CIP), which, in the case of Na_2_CO_3_ solution systems, would be the binary species of NaCO_3_^−^ due to the inability of Na^+^ to complex with CO_3_^2−^ 33. The formation of CIP would weaken the 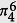 bond of CO_3_^2−^ and lower its force constant, causing the *ν*_1_-CO_3_^2−^ Raman line to shift to low wavenumbers. This hypothesis is supported by experimental results from several earlier studies. By using Na^+^ ion-selective glass electrode potentiometry, Capewell, *et al*.^34^ demonstrated that the association constant of the reaction 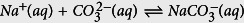 was inversely correlated with the total ionic strength of the solution. Schmidt^14^, on the other hand, noticed the existence of large systematic residuals when attempting to perform a two-peak fitting on the 900–1200 cm^−1^ region of the Raman spectrum of 1.6 M Na_2_CO_3_ solution obtained at 500 °C and 1.3 GPa. He subsequently eliminated the residuals by adopting a three-peak fitting strategy that entailed the assignment of the 1050 cm^−1^ peak to the elusive NaCO_3_^−^ species. However, this method is feasible only at elevated pressure and temperature, where the formation of HCO_3_^−^ and NaCO_3_^−^ is comparatively more favorable from a thermodynamic standpoint^15^. In the current study, we argue that the relative proportion of the NaCO_3_^−^ peak is correlated to the wavenumber shift of the *ν*_1_-CO_3_^2−^ Raman line, the latter of which can therefore be used to evaluate the ionic strength effect.

**Figure 3:**
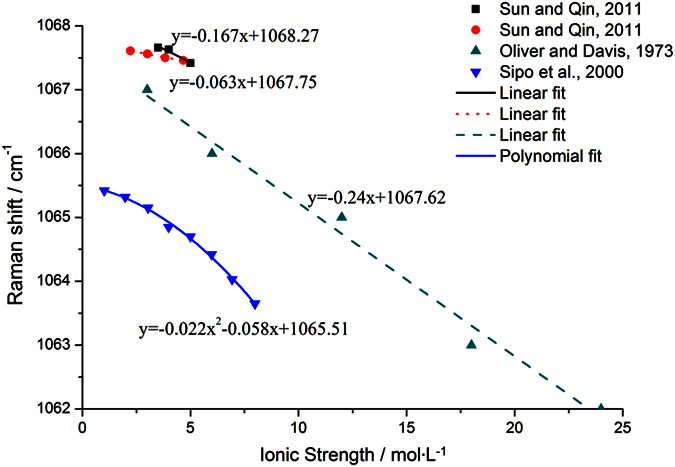
The *ν*_1_*-*CO_3_^2−^ Raman line shifts to low wavenumbers with increasing ionic strength in pure or mixed carbonate sodium solutions (Square: 1 M Na_2_CO_3_ and 0.5–2.0 M NaCl; Round: 0.741–1.556 M Na_2_CO_3_; Triangle: 1–8 M Na_2_CO_3_; Inverted triangle: 0.1 M Na_2_CO_3_ and 0.713–7.681 M NaOH). The relationships between Raman frequencies and ionic strengths are fitted as linear equations for the first three samples and as a quadratic equation for the last sample, respectively. The fitting results indicate that the correlation between *ν*_1_*-*CO_3_^2−^ Raman frequencies and ionic strengths is influenced by anion species in solution systems.

A comparison among the results from different studies, as summarized in Fig. 3, indicated that although the introduction of additional anions always caused the *ν*_1_-CO_3_^2−^ Raman peak to shift toward low wavenumbers, the extent of the shift (∂*ν*_1_/∂*I*)_*P,T*_ varied according to the specific type of anion. Among those examined, Cl^−^ was observed to have a more pronounced effect than the oxygen-containing anions. This was probably due to the fact that oxygen-containing anions tend to form solvent-separated (SSIP) or double solvent-separated ion pairs (2SIP) through hydrogen-bonding with solvent water molecules^35^,^36^; therefore, they are capable of attracting and sequestering a greater number of cations, making them less available for CIP formation with CO_3_^2−^. Unfortunately, the intrinsic complexity of solute-solute and solute-solvent interactions constitutes a formidable barrier for the reliable quantification of the ionic strength effect on Raman frequency shift. To simplify the problem, we took advantage of the fact Cl^−^ has the greatest abundance in various natural environments and generally plays a more significant role than the common oxygen-containing anions in Raman spectroscopy. Meanwhile, given that there have been well-established methods for quantifying salinity via freezing-point depression^37^ or the Raman outline of water^38^, it is feasible to estimate the Cl^−^ concentration in natural fluid systems. Thus, we decided to focus exclusively on the contribution of Cl^−^ to the Raman shift of CO_3_^2−^ in subsequent analyses.

As illustrated in Fig. 2 and Table 2, the slope of Raman shift to pressure for the *ν*_1_-CO_3_^2−^ line was unaffected by the increase in the concentration of NaCl. This, combined with similar findings on pure Na_2_CO_3_ solutions in our previous study, suggested a lack of cross-interaction between pressure and ionic strength^13^. Therefore, the contribution of ionic strength to the Raman frequency shift of Na_2_CO_3_ in solution can be expressed as a simple linear equation. In order to determine the slope of Raman frequency with regard to ionic strength (∂*ν*_1_/∂*I*)_*P,T*_, we opted to follow Sun and Qin^30^’s study on NaCl-Na_2_CO_3_ solution systems based on two considerations: i) The Raman spectra in their study were collected over a relatively long scanning period (30 sec), which we believed should lead to better measurement accuracy; ii) The NaCl-Na_2_CO_3_ solutions used in their study closely resembled the samples that we investigated in composition. Based on their experimental data, we concluded that for pure Na_2_CO_3_ solution, the correlation between its Raman shift and the ionic strength could be expressed as (∂*ν*_1_/∂*I*)_*P,T*_ = −0.0625·Δ*I*, where Δ*I* = *I*−*I*^θ^ = *I*−3.0 (unit: M, hereinafter for ionic strength) and 2.22 ≤ *I* ≤ 4.67 (*I*^θ^ indicates the ionic strength of the standard Na_2_CO_3_ solution at 1 M). Additionally, NaCl-dependent Raman frequency shift was calculated to be −0.167·*I*_*NaCl*_, where 0.5 ≤ *I*_*NaCl*_ ≤ 2.0. One drawback in Sun and Qin’s study was the narrow range of ionic strength that they investigated; as a result, the maximum contribution of ionic strength to *ν*_1_-CO_3_^2−^ Raman frequency shift was estimated to be around 0.2 cm^−1^, which is roughly on the same level as the system error. Still, it has been suggested elsewhere that this linear relationship could be applied to solution systems with much greater ionic strength (up to *I* = 24.0)^19^.

Several other groups have quantified the slope of *ν*_1_-CO_3_^2−^ Raman line shift with temperature. For example, its temperature dependence at 1 GPa and in the range of 100–275 °C was calculated to be approximately −0.041 cm^−1^ ∙ °C^−1^ 21 (Table 4). However, due to the tendency of quartz dissolution in superheated aqueous solutions^39^,^40^, it is impossible to eliminate the quartz contamination on the estimation of the thermal dependence of the *ν*_1_-CO_3_^2−^ Raman spectrum if quartz were used as the pressure indicator in HDAC. Frantz’s experiment design involved the use of a hydraulic pump to control the pressure of the sample chamber, which offered several advantages over the conventional DAC system^41^. First, the pump provided enhanced pressure stability during each spectral analysis, with a maximum fluctuation of 10 bars, whereas DAC is generally incapable of maintaining an isobaric condition when the sample chamber is heated. Second, Frantz measured the pressure of the system with a pressure meter, which could lead to better accuracy and more reliable performance. It should also be noted that Frantz conducted the Raman spectroscopic study over a wide temperature range (from room temperature to 550 °C). Therefore, we relied on Frantz^15^’s data, which reported a global value of −0.0446 cm^−1^ ∙ °C^−1^ for the temperature dependence of the Raman shift ((∂*ν*_1_/∂*P*)_*T*_. Table 4).

The pressure- and temperature-dependence of Raman frequency have also been scrutinized in various other solid- and solution-based systems. Frantz^15^ reported that the *ν*_1_-CO_3_^2−^ Raman line shifted toward low wavenumbers with increasing temperatures at a rate (∂*ν*_1_/∂*T*)_*P*_ that was largely pressure-independent. The *ν*_1_-SO_4_^2−^ Raman line was shown to shift toward low wavenumbers with increasing pressure at a linear rate (∂*ν*_1_/∂*P*)_*T*_ of 0.19 cm^−1^ ∙ GPa^−1^ 8. Similarly, the Raman frequency of quartz and sphalerite exhibited an inverse linear relationship with pressure at a slope (∂*ν*_1_/∂*P*)_*T*_ that was mostly uniform within the temperature range tested^5^,^27^. Previous empirical fitting equations in Raman studies demonstrated that the cross-derivatives of the frequency shifts of Raman lines to the pressure and the temperature could be assumed as zero^42^. Therefore, we reasoned that the thermal effect can be expressed as a simple linear relationship between temperature and the Raman of *ν*_1_-CO_3_^2−^ wavenumber with no cross-interaction with the pressure effect.

No systematic analyses have been performed to investigate whether there is interplay between temperature and ionic strength in their influence on Raman spectra of Na_2_CO_3_ solutions. To circumvent this problem, we compared the isobaric temperature slopes (∂*ν*_1_/∂*T*)_*P*_ for the Raman line of SO_4_^2−^ separately obtained by Schmidt^9^ and Qiao, *et al*.^8^, both of whom measured the spectra in DAC with quartz as the pressure gauge. In Schmidt^9^’s study using a solution containing 1.54 M Na_2_SO_4_, the slope was calculated to be −0.0231 cm^−1^ ∙ °C^−1^ at 322 MPa and in the range of 21–400 °C exhibited (R^2^ = 0.9985), whereas Qiao, *et al*.^8^ reported a result of −0.0224 cm^−1^ ∙ °C^−1^ at 340 MPa and 100–250 °C for 1 M solution. However, according to Rull, *et al*.^43^’s estimation, the presence of an additional 0.54 M SO_4_^2−^ would cause a Raman frequency shift of 0.2 cm^−1^. It can be seen that the difference between the two slopes, which represents the interaction between the temperature effect and the ionic strength effect on Raman frequency, is almost negligible compared to the Raman shift of SO_4_^2−^ generated from the increase of ionic strength alone. Similarly, this difference was also rather insignificant when compared to the change of Raman frequency caused by temperature variation. Therefore, we tentatively concluded that given the instrumental uncertainties in typical high-pressure Raman spectroscopic measurement, the cross-interaction between temperature and ionic strength could be safely omitted when estimating their impact on the Raman frequency of solutes.

Based on our experiment results and discussions detailed above, the empirical equation describing the correlation among pressure, temperature, ionic strength and the *ν*_1_-CO_3_^2−^ Raman characteristic frequency at 1066 cm^−1^ in aqueous solution can be expressed as follows:


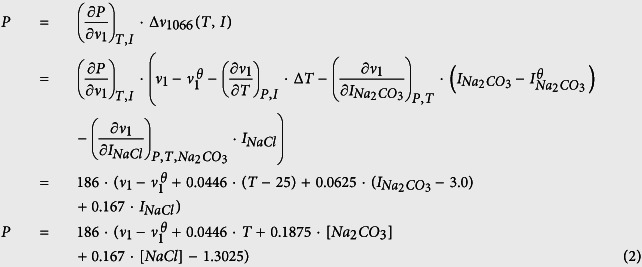


where *P, T*, and *I* represent the pressure (MPa), temperature (°C), and the ionic strength of the solution system (M), respectively; [X] indicates the concentration of the solute X (M); *v*_1_ and 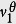 denote the *ν*_1_-CO_3_^2−^ Raman wavenumber measured under experimental conditions and standard conditions (as defined by 0.1 MPa, 25 °C, and 1.0 M Na_2_CO_3_ solution), respectively (cm^−1^).

The standard error of Eq. (2) is derived from the fitting uncertainties of temperature, the concentration of Na_2_CO_3_, and the concentration of NaCl, whose contributions are ±0.13, ±0.03, and ±0.04 (all in cm^−1^), respectively. Since there is no cross-interaction between any two of these three variables, the overall standard error of Eq. (2) should be ±0.20 cm^−1^, which corresponds to a deviation of 37 MPa for the calculated pressure. On the other hand, the maximum standard error for the linear fitting equation shown in Table 2 is 18 MPa. Therefore, the theoretical maximum error generated from the calculation of pressure via Eq. (2) is less than 37 MPa. Obviously, the ionic strength effect can also be incorporated into 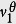 to afford 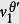, which denotes the Raman wavenumber of CO_3_^2−^ in the fluid sample (as opposed to in 1.0 mol·L^−1^ Na_2_CO_3_ solution) under the standard *P-T* conditions. As a result, Eq. (2) can be rewritten as below:


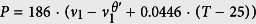


The equation that we have developed in this study has several practical ramifications for pressure determination in various natural and artificial fluid inclusions. To begin with, the equation contains additional terms for quantifying the impact of ionic strength on the Raman shift of Na_2_CO_3_. As an example, it can be inferred from the equation that the presence of 2 M NaCl in the solution can result in a deviation of 62 MPa in the calculated pressure, which exceeds the maximum theoretical error of 37 MPa mentioned above. This highlights the importance of incorporating ionic strength as an additional variable when calculating the internal pressure of various fluid systems. Natural fluid inclusions frequently contain daughter minerals that are formed by cooling and depressurization as a result of stratum exhumation during following geological evolution. When these fluids were analyzed in high *P-T* experiment settings, the daughter minerals could re-dissolve, increasing the ionic strength of the resultant solution and consequently causing pressure calculation errors. The impact of mineral contamination on pressure measurement could potentially be quantified and even corrected based on the strategy that we proposed in this study.

In addition, the equation also allows one to evaluate the impact of excess salinity on the accuracy of pressure measurement at different depths. A previous study conducted by Mao *et al*. estimated the overall relative uncertainty of ruby to be around ±6%^44^. Therefore, the systematic offset resulting from the ionic strength of a 2 M NaCl solution (~ 10.5 wt%) would cause a similar level of relative uncertainty when the internal pressure of the fluid inclusion to be measured is around 1 GPa (an uncertainty of 6%), which corresponds to a depth of ~30 km below ground. Obviously, the contribution of this error would become substantially more significant when the fluid inclusion is found at a shallower depth, at which point any meaningful pressure measurement would be rendered impractical. This poses a particular challenge to the geological characterization of sedimentary basins located within a depth of 10 km to the ground level, which are often more enriched with fluids compared to magma chambers or subduction zones in the upper mantle or below. Raman measurements performed on these fluid systems often required stringent pressure calibration and higher spectral resolution. For example, the density and pressure of CH_4_-H_2_O system can be calculated from the Raman shift *v*_1_-CH_4_ and *P-V-T* equations. Increased resolution of the Raman spectrum (±0.3 cm^−1^) was shown to result in a lower uncertainty of the pressure calculation (down to 3 MPa)^29^, which is acceptable even at a depth of 3 km (~30 MPa of hydrostatic condition).

The physicochemical properties of aqueous fluids under high *P-T* conditions are indispensable for investigating geochemical behaviors at great depths^2^. Because of the inherent complexity of natural fluid inclusions, aqueous solutions of Na_2_CO_3_ or Na_2_SO_4_ have often been used as simplified model systems^9^,^10^,^14^. However, when performing laboratory studies on these solutions, the high *P-T* conditions can cause conventional crystal*-*based pressure gauges to partially dissolve, leading to altered fluid compositions. For example, the solubility of quartz increases dramatically from 0.78 wt% at 500 °C and 5 kbar to 12.56 wt% at 900 °C and 10 kbar^45^. This problem can be addressed by replacing the conventional pressure gauges with a solute-based barometer when the high *P-T* experiments are performed under conditions that could potentially trigger undesirable side reactions or cause the crystal calibrants to dissolve.

In short, the current study supports that alterations in fluid salinity could have a significant impact on ionic interaction^9^, mineral solubility^46^, as well as the *P-V-T* diagrams of various aqueous systems^47^. Since NaCl is the most significant contributor to the ionic strength in natural fluid inclusions, we argue that it should be considered as a necessary, or even essential, component in laboratory investigations of artificial electrolytic solutions, such as Na_2_CO_3_- and Na_2_SO_4_-based systems, in order to more accurately characterize the physicochemical behaviors of aqueous fluids at natural geological settings. Meanwhile, we are convinced that our carbonated-based barometer adds to the current arsenal of pressure sensors for *in-situ* spectroscopic observation under high *P-T* conditions.

## Methods

In order to probe the effect of Cl^−^ on the Raman spectrum of CO_3_^2−^, three solutions consisting of 2 M Na_2_CO_3_ with three concentrations of NaCl (0.20, 0.42, and 0.92 M) were prepared in distilled water and each set to a final volume of 50 mL in a volumetric flask. High-pressure experiments were conducted in a moissanite anvil cell modified from a diamond anvil cell (DAC)^48^. The sample chamber comprised a 0.3 mm diameter hole drilled in the center of a 0.5 mm thick stainless steel gasket and flanked between the two anvils. A small piece of quartz was placed as an internal pressure gauge in the sample chamber, where the aqueous sample solution was also enclosed. The Raman spectra of the quartz and the sample were separately measured in the wavenumber range of 50–4000 cm^−1^ using a Raman microspectrometer (with a spectral resolution of ±1 cm^−1^, Renishaw system RM-1000, Renishaw Group, Gloucestershire, United Kingdom) equipped with a charged coupled detector and a Leitz 20X working objective. A 25 mW argon laser (514.5 nm) was used for sample excitation. The slit width and the collection time were set to 50 μm and 10 s, respectively. All measurements were performed at ambient temperature (approximately 22 °C) and under the pressure range of 100–1600 MPa. A three-minute interval was introduced immediately after each pressure change between two consecutive measurements in order for the sample to regain equilibrium. Peakfit4 was used to fit the Raman spectrum of quartz in the range of 300–600 cm^−1^ and of CO_3_^2−^ in the range of 1000–1200 cm^−1^ into Gaussian peaks, from which spectrometric parameters such as wavenumber were derived.

The internal pressure in the experimental volume inside the diamond anvil cell was determined using the shift of the most intense Raman band of quartz near 464 cm^−1^ using the calibration equation^5^:


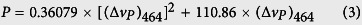


where (∆*ν*_*P*_)_464_ indicates the pressure-induced frequency shift for the characteristic Raman peak of quartz from the standard value of 464 cm^−1^ measured under ambient conditions. It should be noted that equation (3) would yield a measurement uncertainty of ± 50 MPa and its application should be limited to 0 < (∆*ν*_*P*_)_464_ ≤ 20 cm^−1^ and *P* < 2.0 GPa.

## Additional Information

**How to cite this article**: Wu, J. *et al*. The influence of ionic strength on carbonate-based spectroscopic barometry for aqueous fluids: an *in-situ* Raman study on Na_2_CO_3_-NaCl solutions. *Sci. Rep.* **6**, 39088; doi: 10.1038/srep39088 (2016).

**Publisher's note:** Springer Nature remains neutral with regard to jurisdictional claims in published maps and institutional affiliations.

## Figures and Tables

**Table 1:** Raman shifts of *ν*_1_ *-*CO_3_^2−^ with increasing pressure based on three Na_2_CO_3_-NaCl solutions.

No. 1		No. 2		No. 3	
*P*/MPa	Δ*ν*_1066_/cm^−1^	*P*/MPa	Δ*ν*_1066_/cm^−1^	*P*/MPa	Δ*ν*_1066_/cm^−1^
122	0.8	122	0.4	134	0.5
145	1.0	145	0.5	190	0.7
156	1.2	201	0.9	257	0.5
246	1.7	190	1.0	540	2.5
336	1.8	336	1.4	632	2.8
678	3.5	483	2.3	678	3.2
771	4.3	759	3.6	887	4.0
957	5.2	724	3.7	968	4.9
1027	5.6	817	4.2	1109	5.8
1050	6.2	1027	5.7	1133	5.7
1168	6.0	1074	5.8	1216	6.7
1347	7.7	1204	5.8	1251	6.5
		1263	6.6	1394	6.3
		1323	6.7	1538	7.5
		1454	7.5		

**Table 2:** Quantification of the pressure influence on *ν*_1_*-*CO_3_^2−^ Raman shift in three Na_2_CO_3_-NaCl solutions.

[Na_2_CO_3_]	[NaCl]	Fitting equation (*P* = aΔν_1066_ + b)		R^2^	Standard error
mol·L^−1^	mol·L^−1^	Slope (MPa/cm^−1^)	Intercept (MPa)		MPa
2.01	0.20	185(6)	29(27)	0.988	14
2.00	0.42	187(4)	44(19)	0.993	11
1.99	0.92	185(8)	92(36)	0.978	18

*P* is pressure in MPa. Δν_1066_ is the relative *ν*_1_*-*CO_3_^2−^ Raman shift under pressure, and its value is between 0 and 10 cm^−1^. The numbers in the brackets indicate the standard deviations (2σ) for the slope and the intercept. The standard errors (MPa) are calculated with the data in Table 1 and the fitting equations in Table 2.

**Table 3:** Quantification of the ionic strength influence on *ν*_1_ *-*CO_3_^2−^ Raman shift at ambient conditions based on previous references.

Sources	[CO_3_^2−^]	[Cl^−^]	∂ν/∂*I*	R^2^	Standard error
	mol·L^−1^	mol·L^−1^			cm^−1^
Oliver and Davis^19^	1.0−8.0^a^	0	−0.24	0.987	n.d.
Sun and Qin^30^	0.768−5.12^a^	0	−0.181	0.998	n.d.
	0.741 −1.56^b^	0	−0.063	0.993	0.033
	1.0^b^	0.5−2.0	−0.167	0.95	0.041
Sipos *et al*.^20^	0.1^b^	0.713−7.68*	−0.044*I*–0.058	0.996	n.d.
	0.1^b^	<4.7	~ −0.113	n.d.	n.d.

^a^Potassium carbonate solution.

^b^Sodium carbonate solution.

^*^Chloride anion is replaced by hydroxyl anion; n.d. indicates that the value is “not determined”.

**Table 4:** Quantification of the temperature influence on *ν*_1_ *-*CO_3_^2−^ Raman shift based on previous references.

Sources	[CO_3_^2−^]	*P*/MPa	Fitting equation (ν_1066_ = a·*T* + b)		R^2^	Standard error
	mol·L^−1^		∂ν/∂*T*	Intercept		cm^−1^
Frantz^15^^ ^a	1.0	100	−0.0452	1072.4	0.997	0.14
	1.0	150	−0.0446	1072.7	0.997	0.13
	1.0	200	−0.0440	1072.9	0.997	0.12
Martinez *et al*.^21^^ ^b	2.0	1000	−0.0411	1072.8	0.97	n.d.

ν_1066_ is in cm^−1^. It is the carbonate Raman shift under a constant pressure *P* (MPa). T is temperature in °C, and its value ranges ^a^ from 24 to 550 °C, and ^b^ from 100 to 275 °C. The standard deviations for the slope and the intercept are not determined.

^a^Potassium carbonate solution.

^b^Sodium carbonate solution; n.d. indicates that the value is “not determined”.
